# Comparison of deep or moderate neuromuscular blockade for thoracoscopic lobectomy: a randomized controlled trial

**DOI:** 10.1186/s12871-018-0666-6

**Published:** 2018-12-21

**Authors:** Xiao-feng Zhang, De-yuan Li, Jing-xiang Wu, Qi-liang Jiang, Hong-wei Zhu, Mei-ying Xu

**Affiliations:** Department of Anesthesiology, Shanghai Chest Hospital, Shanghai Jiaotong University, 241 huaihai west road, Shanghai, 200030 China

**Keywords:** Thoracoscopic surgery, Neuromuscular blockade, Surgeon satisfaction

## Abstract

**Background:**

Laparoscopic surgery typically requires deep neuromuscular blockade (NMB), but whether deep or moderate NMB is superior for thoracoscopic surgery remains controversial.

**Methods:**

Patients scheduled for thoracoscopic lobectomy under intravenous anesthesia were randomly assigned to receive moderate [train of four (TOF) 1–2] or deep NMB [TOF 0, post-tetanic count (PTC) 1–5]. Depth of anesthesia was controlled at a Narcotrend rating of 30 ± 5 in both groups. The primary outcome was the need to use an additional muscle relaxant (cisatracurium) during surgery. Secondary outcomes included surgeon satisfaction, recovery time of each stage after drug withdrawal [time from withdrawal until TOF recovery to 20% (antagonists administration), 25, 75, 90, 100%], blood gas data, VAS pain grade after extubation, the time it takes for patients to begin walking after surgery, postoperative complications and hospitalization time. Results were analyzed on an intention-to-treat basis.

**Results:**

Thirty patients were enrolled per arm, and all but one patient in each arm was included in the final analysis. Among patients undergoing moderate NMB, surgeons applied additional cisatracurium in 8 patients because of body movement and 5 because of coughing (13/29, 44.8%). Additional cisatracurium was not applied to any of the patients undergoing deep NMB (*p* < 0.001). Surgeons reported significantly higher satisfaction for patients undergoing deep NMB (*p* < 0.001, Wilcoxon rank sum test). The mean difference between the two groups in the time from withdrawal until TOF recovery of 25% or 90% was 10 min (*p* < 0.001). The two groups were similar in other recovery data, blood gas analysis, VAS pain grade, days for beginning to walk and mean hospitalization time.

**Conclusions:**

Deep NMB can reduce the use of additional muscle relaxant and increase surgeon satisfaction during thoracoscopic lobectomy.

**Trial registration:**

Chinese Clinical Trial Registry, ChiCTR-IOR-15007117, 22 September 2015.

## Background

Sufficient muscle relaxation is mandatory for most surgical procedures, and particularly for minimally invasive surgery [1–3]. For laparoscopic surgery, deep neuromuscular blockade (NMB) can decrease the need for high pneumoperitoneum pressure [4, 5] and prevent sudden abdominal contractions, thereby reducing the risk of respiratory and circulatory complications [6]. However, Kopman AF and Naguib M noted that the use of low-pressure pneumoperitoneum was often associated with a substantial reduction in visibility and in available working space, and these factors could negatively affect patient outcome in terms of increased difficulty in dissection and might result in increased risk of organ injury and operating time [7, 8]. There is still a controversy regarding the need and clinical benefit of maintaining deep neuromuscular blockade for routine laparoscopic surgery.

Whether deep NMB can similarly benefit patients undergoing thoracoscopic surgery is unclear. On one hand, it may seem unnecessary because the ribcage provides thoracic support and one-lung ventilation usually provides a satisfactory surgical field. On the other hand, thoracoscopic surgeries involve areas adjacent to major blood vessels and can trigger intraoperative body movement, cough, and diaphragm movement [9], the diaphragm is the most resistant muscle to NMBAs, movement of the diaphragm can interfere with the surgical procedure. The cough reflex, which is mediated by rapidly adapting receptors in the throat and protuberantia, is suppressed using muscle relaxants to inhibit signaling at neuromuscular junctions [10]. One study has suggested that maintaining PTC ≤ 5 can inhibit response to carinal stimulation and prevent bucking and coughing during surgical procedures, despite total abolition of the abductor pollicis muscle TOF response [11]. Another study suggested that PTC ≤ 5 is required to achieve deep NMB of the diaphragm [12].

Here we compared deep and moderate NMB for their ability to reduce requirement of additional muscle relaxant and improve surgeon’s assessment in patients undergoing thoracoscopic lobectomy. An anaesthesiologist blinded to patient allocation was responsible for collecting perioperative data. We expected that deep NMB would be superior because of its demonstrated ability to reduce peak pressure and plateau pressure in the airway as well as improve lung compliance and peripheral oxygen saturation during one-lung ventilation [13].

## Methods

### Patients

This is a single-center, randomized, controlled trial approved by the Ethics Committee of research institution (#KS1520, Date of approval: 2015/8/17) and written informed consent was obtained from all subjects participating in the trial. Trial registration: Chinese Clinical Trial Registry, ChiCTR-IOR-15007117, 22 September 2015.

The trial was carried out between October 2015 and July 2016 in the Department of Anesthesiology. Patients had to satisfy the following inclusion criteria: (1) age of 18–65 years, (2) elective thoracoscopic lobectomy, (3) American Society of Anesthesiologists (ASA) classification of I or II, and (4) body mass index (BMI) of 18–25 kg/m^2^. Patients were excluded if they had history of diabetes, viral hepatitis, asthma, glaucoma, neuromuscular disease, or if airway difficulties were anticipated. Patients were also excluded if they had hepatic or renal dysfunction, defined as one of the following: ALT > 40 U/L, AST > 40 U/L, SCr > 133 μmol/L, or total bilirubin > 30 μmol/L (Because these factors can affect the metabolism of analgesic and sedative drugs, glaucoma is a contraindication for the use of muscle relaxants antagonists).

### Randomization

Patients were allocated to deep or moderate NMB groups using random numbers generated by computer (SAS Institute, Cary, NC, USA) and concealed in opaque envelopes. An anaesthesiologist aware of patient allocation monitored depth of anesthesia and NMB. A different anaesthesiologist blinded to patient allocation was responsible for airway management, intubation, arteriovenous puncture, monitoring of vital signs and temperature and blood gas analysis. Patients and surgeons were blinded to group assignment. The same group of surgeons performed all procedures for both groups of patients.

### Surgery

Patients underwent lobectomy involving standard thoracoscopy as described [14]. Lymph nodes were sampled via a minimally invasive procedure in patients with lesions < 2 cm, whereas lymph nodes were systemically dissected in patients with lesions ≥2 cm [15, 16]. Lymph nodes were sampled based on vision and touch: any nodes suspected of harboring cancer were removed and submitted for histopathology analysis. Systemic lymph node dissection involved continuous, complete dissection of mediastinal lymph nodes along with surrounding adipose tissue. Lymph nodes in groups 2, 4, 7, and 10 were routinely removed from patients with cancer in the right lung, while lymph nodes in groups 5, 6, 7, and 10 were routinely removed from patients with cancer in the left lung.

### Anesthesia

Surgery was conducted under total intravenous anesthesia without preoperative medication. Anesthesia was conducted using propofol TCI (4 μg/ml) and sufentanil (0.7 μg/kg). NMB calibration was carried out under ventilation with a face mask after patients lost consciousness. Cisatracurium (0.2 mg/kg, bolus injection) was given after TOF stabilization. When TOF reached 0, patients were intubated with a double-lumen endotracheal tube for single lung ventilation under bronchofiberscope guidance. Ventilation was carried out with pure oxygen and adjusted to maintain end-tidal carbon dioxide pressure at 35–40 mmHg.

Anesthesia was maintained with propofol TCI (2–3 μg/ml) and dexmedetomidine (0.4 μg/kg/min) to maintain the Narcotrend (Germany) rating at 30 ± 5. Sufentanil was continued at 5–10 μg/h. Dezocine (5 mg) and ramosetron (0.6 mg) were given 15 min before the end of the operation. After the last skin suture, propofol and dexmedetomidine were discontinued.

Given the need for continuous muscle relaxant monitoring and in light of the stability of patient baseline parameters, we did not move patients until TOF recovered to 90%. The double-lumen tube was removed in the operating room and patients were given mask oxygen (FiO_2_ = 40%), observed closely for 10 min and transferred to the post-anesthesia care unit. When blood gas results were normal, patients were transferred to the ward or the intensive care unit.

### Monitoring of NMB depth

The patient was operated on the lateral position, with one arm outspread on a pallet, this arm and hand can be used for monitor of NMB. NMB was monitored using TOF-Watch-SX (MSD BV, Oss, the Netherlands). Electrodes were placed over the ulnar nerve proximal to the wrist at a distance of 3–6 cm. The TOF-Watch applies an electrical stimulus to the ulnar nerve and measures contractions of the adductor pollicis muscle through a sensor attached to the tip of the thumb. Deep NMB was defined as 1 ≤ PTC ≤ 5 [17].

Tetanic stimulation was applied to the ulnar nerve (50 Hz for 5 s) once the patient was unconscious, then the TOF-Watch was calibrated. After TOF stimulation remained stable for at least 3 min, stimulation was switched to the TOF stimulation pattern (50 mA, 0.2 ms pulse duration, 2 Hz). If the TOF ratio differed by 10%, the TOF-Watch was recalibrated. TOF was monitored every 15 s, and PTC every 3 min.

Muscle relaxant infusion started when PTC reached 1 in the deep NMB group or 8 in the moderate NMB group. Initial rate of cisatracurium infusion was 0.1 mg/kg/h. The infusion rate was adjusted in increments of 0.01 mg/kg/h in order to maintain PTC at 1–5 in the deep NMB group or TOF at 1–2 in the moderate NMB group. Core temperature was controlled at 36–37 °C; skin surface temperature was maintained above 32 °C using a warm air blower. Additional cisatracurium (0.05 mg/kg) was given when requested by the surgeon, regardless of the reasons.

Infusion of muscle relaxants was stopped upon placement of the chest drainage tube. When TOF recovered to 20%, neostigmine (0.02 mg/kg) and atropine (0.02 mg/kg) were injected. In all patients, neuromuscular monitoring continued until the TOF ratio reached 90%.

### Outcomes

The primary outcome was the use of additional cisatracurium during surgery. One secondary outcome was the surgeon’s assessment with how well he was able to perform the procedure without interference from coughing, bucking, muscle contractions or lung movement. The four-point scale [18] was (1) extremely poor, (2) poor, (3) good, and (4) excellent (Online Resource 1). Other secondary outcomes included time needed for TOF recovery to 25–75% upon drug discontinuation, interval between antagonism (TOF = 20%) and recovery of TOF = 90%, time from withdrawal until TOF recovery to 25% or 90%, time from end of surgical procedure until full NMB recovery, blood gas data, VAS pain grade after extubation, hospitalization time and the time it took for patients to begin walking after surgery. The operative events [body movement, coughing, and breathing against the ventilator (with the aid of airway pressure monitoring and capnography)] and postoperative complications (bronchial anastomotic fistula, intrathoracic hemorrhage, atelectasis, pneumonia, respiratory insufficiency, arrhythmia, cardiac tamponade, heart failure) were recorded. The occurrence of postoperative complications were evaluated by the blinded doctor-in-charge before the patient was discharged from the hospital.

### Sample size calculation

We calculated (software: PASS 11 [NCSS, Kaysville, UT, USA]) a minimum requirement of 20 subjects in each arm of the study based on the idea that 40% of patients in the moderate NMB group and none of the patients in the deep NMB group would receive additional muscle relaxant [19] and based on the criteria of α at 0.05 (one-sided) and 1-β at 90%. Therefore we planned to enroll 30 patients in each arm in order to compensate for the possibility of equipment failure and protocol violations.

### Statistics

Statistical analyses were performed using SAS 9.2 (SAS Institute, Cary, NC, USA) on an intention-to-treat basis. Numbers of surgeon requests for additional muscle relaxant, incidence of intraoperative events and postoperative complications were compared between the two groups using Fisher’s exact probability method. Surgical characteristics and VAS pain grade were compared between the two groups using the Wilcoxon rank sum test. Differences in baseline and recovery characteristics were compared using Student’s *t* test when the data were normally distributed or using the Wilcoxon rank sum test when the data were skewed. Results associated with a two-sided *P* < 0.05 were considered statistically significant.

## Results

### Patients

**Fig. 1:**
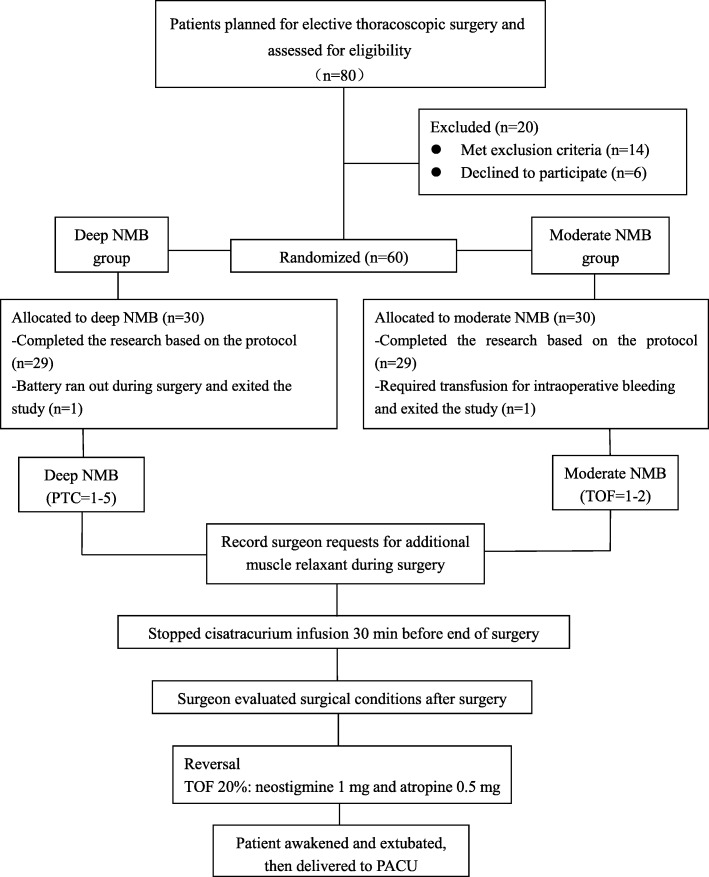
Flow chart. Note: NMB: Neuromuscular blockade. TOF: Train of four. PTC: Post tetanic count. PACU: Postanesthesia care unit. After extubation, the patient was transferred to the PACU and the vital signs were observed

Of the 80 subjects assessed for eligibility, 6 declined to participate and 14 were excluded for the following reasons: hepatic dysfunction (*n* = 2), history of diabetes (7), viral hepatitis (2), asthma (1), and anticipated airway difficulties (2). Of the 60 patients finally enrolled, one was excluded from the deep NMB group because the battery ran out during muscle relaxant monitoring, and one was excluded from the moderate NMB group because of intraoperative bleeding and sent to ICU intubated after surgery. Thus 58 patients were included in the intention-to-treat analysis (Fig. 1).

Patient characteristics and surgical information are summarized in Tables 1 and 2. The two groups were comparable in age, gender composition, surgical site, TNM stage, tumor size, number and metastasis of lymph nodes, operation time, anesthesia depth, and dosage of anesthetics.

**Table 1:** Patient characteristics

	Moderate group (*n* = 29)	Deep group (*n* = 29)
Age, mean(SD), y	56.86(8.25)	56.59(9.70)
Weight, mean(SD), kg	59.66(8.28)	62.59(7.98)
Height, mean(SD), m	1.64(0.08)	1.65(0.07)
BMI, mean(SD)	22.56(2.82)	22.90(2.24)
Women, No. (%)	17(58.62)	16(55.17)
Left lobectomy, No. (%)	11(37.93)	12(41.38)
Central lung cancer, No. (%)	13(44.83)	13(44.83)
TNM stage, No. (%)		
Ia	25(86.21)	22(75.86)
Ib	0(0)	2(6.90)
IIa	4(13.79)	4(13.79)
IIb	0(0)	1(3.45)
tumor size, No. (%), cm		
<2	19(65.52)	19(65.52)
≥2	10(34.48)	10(34.48)
lymph node retrieved number, No. (%)		
<10	12(41.38)	11(37.93)
≥10	17(58.62)	18(62.07)
lymph node metastasis, No. (%)	4(13.79)	5(17.24)

*SD* standard deviation. *BMI* body mass index

**Table 2:** Basic surgical information

	Mean(SD)	
	Moderate group (*n* = 29)	Deep group (*n* = 29)
Anesthesia time, min	93.38(21.07)	99.41(33.09)
Operation time, min	78.79(21.10)	84.10(34.93)
Nacrotrend	30.51(0.53)	30.80(0.68)
Propofol, mean(QR), mg/kg/h	10.25(10.09,10.42)	10.20(10.05,10.37)
Sufentanil, μg/kg/h	0.60(0.10)	0.58(0.16)
Cisatracurium, mg/kg/h	0.07(0.02)	0.12(0.02)

Narcotrend refers to the depth of anesthesia monitoring and was maintained at a target value of 30 ± 5

The average rate of infusion of cisatracurium was 0.07 ± 0.02 mg/kg/h in the moderate NMB group and 0.12 ± 0.02 mg/kg/h in the deep NMB group. These values are within the range of 0.06–0.12 mg/kg/h recommended by the Muscle Relaxant Expert Consensus in 2013 [20].

**Fig. 2:**
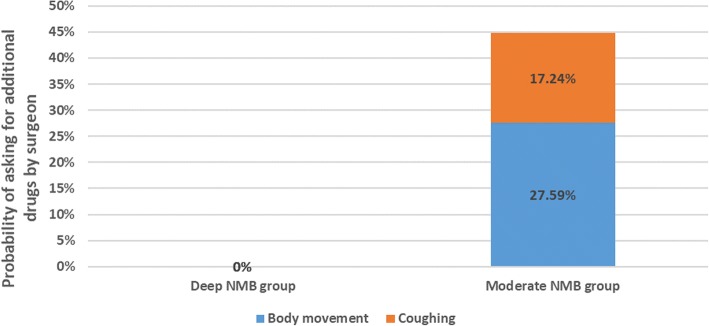
Use of additional muscle relaxant during surgery. Note: Fisher’s exact probability method, *P* < 0.001

Coughing and diaphragm muscle mobility occurred in 10.34% (6/58) of patients during intubation. The average interval between cisatracurium administration during anesthesia induction and recovery of PTC = 1 was 43.33 ± 5.53 min. Among patients in the moderate NMB group, additional muscle relaxant was requested by the operating surgeon for 8 patients due to body movement and 5 due to coughing. Additional relaxant was not requested for any patients in the deep NMB group (44.8% vs 0.0%, *P* < 0.001; Fig. 2).

**Fig. 3:**
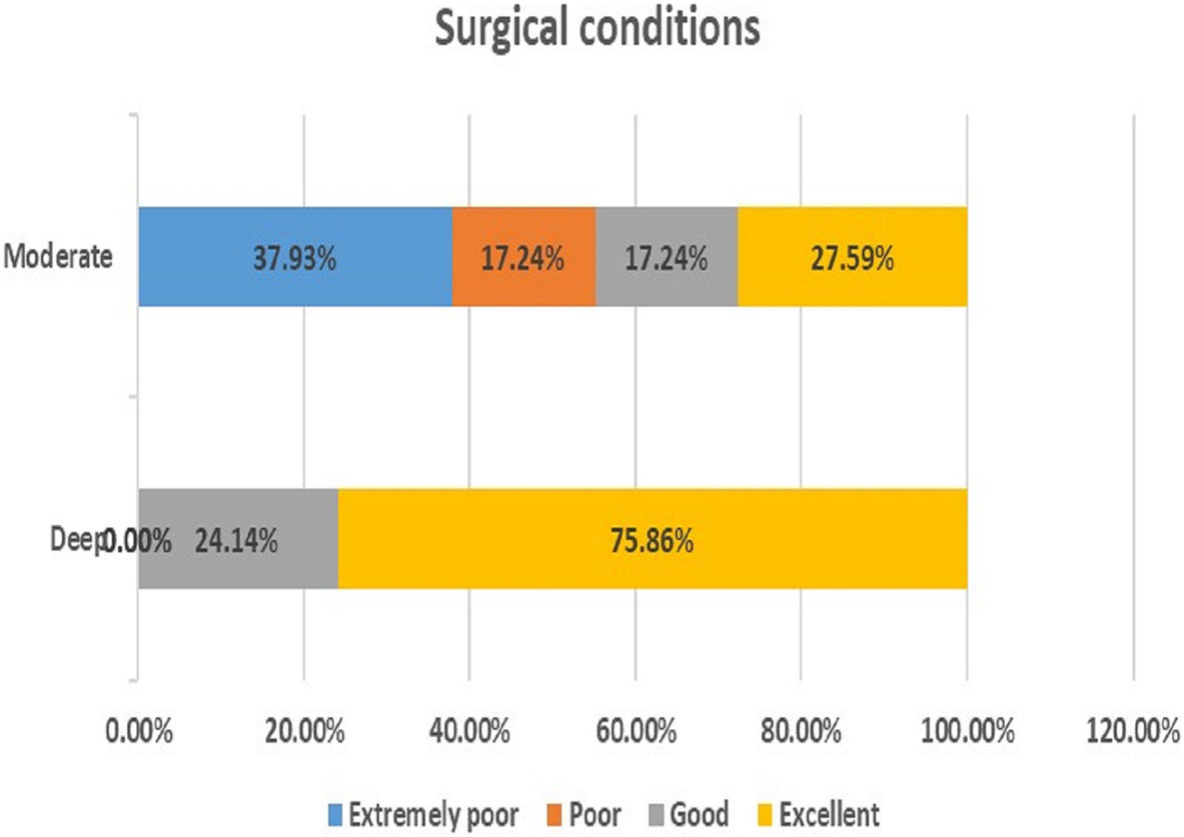
Surgeon-reported satisfaction with operating conditions, based on a four-point scale. Note: Wilcoxon rank sum test, Z = − 4.38, *P* < 0.001

Using a four-point scale, surgeons rated operating conditions for all patients in the deep NMB group as “good” or “excellent”, while they rated conditions as “poor” or “very poor” for 55.17% of patients in the moderate NMB group (Z = − 4.38, *P* < 0.001; Fig. 3).

**Fig. 4:**
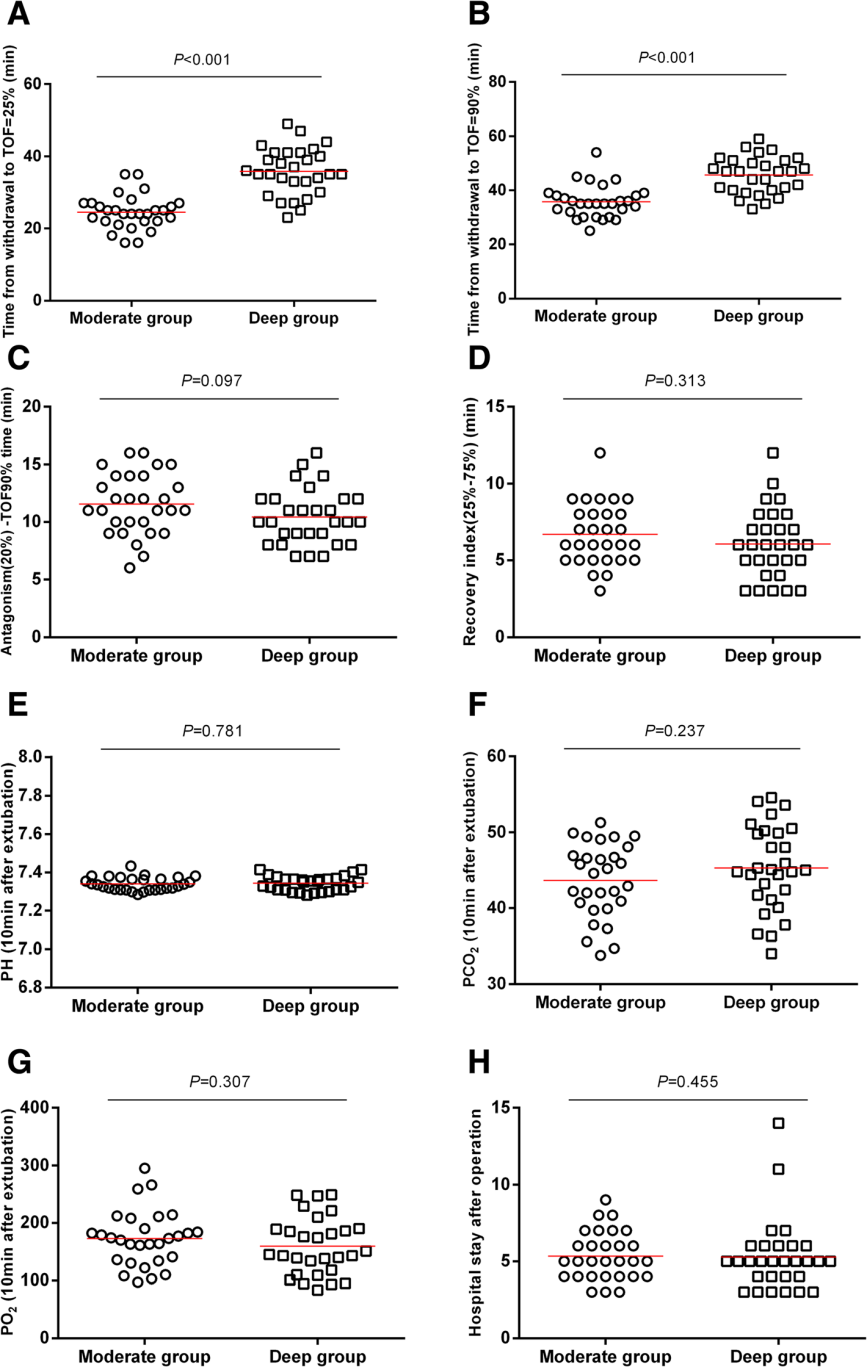
Intervals to achieve different steps in recovery. Note: **a** Interval between withdrawal and recovery of TOF = 25%. The two groups differed significantly (*P* < 0.001). **b** Interval between withdrawal and recovery of TOF = 90%. **c** Interval between antagonism and recovery of TOF = 90%. **d** Interval between recovery of TOF = 25% and recovery of TOF = 75%. **e** Post-extubation pH. **f** PCO_2_ after extubation for 10 min. The two groups were similar. **g** PO_2_ value after extubation for 10 min. The two groups were similar. **h** Length of hospitalization after surgery

The two patient groups showed a similar interval between antagonism (TOF = 20%) and recovery of TOF = 90% (Fig. 4c), as well as similar time to TOF recovery of 25–75% (Fig. 4d). the time from end of surgical procedure until full NMB recovery was 35.16 ± 9.34 min in the moderate NMB (TOF 1–2) group and 45.62 ± 6.81 min in the deep NMB (PTC 1–5) group (*P* < 0.001). The mean difference between the two groups in the time from withdrawal until TOF recovery to 25% (Fig. 4a) or 90% (Fig. 4b) was 10 min. The two groups were similar in blood gas analysis and mean hospitalization time (Fig. 4e-h).

**Fig. 5:**
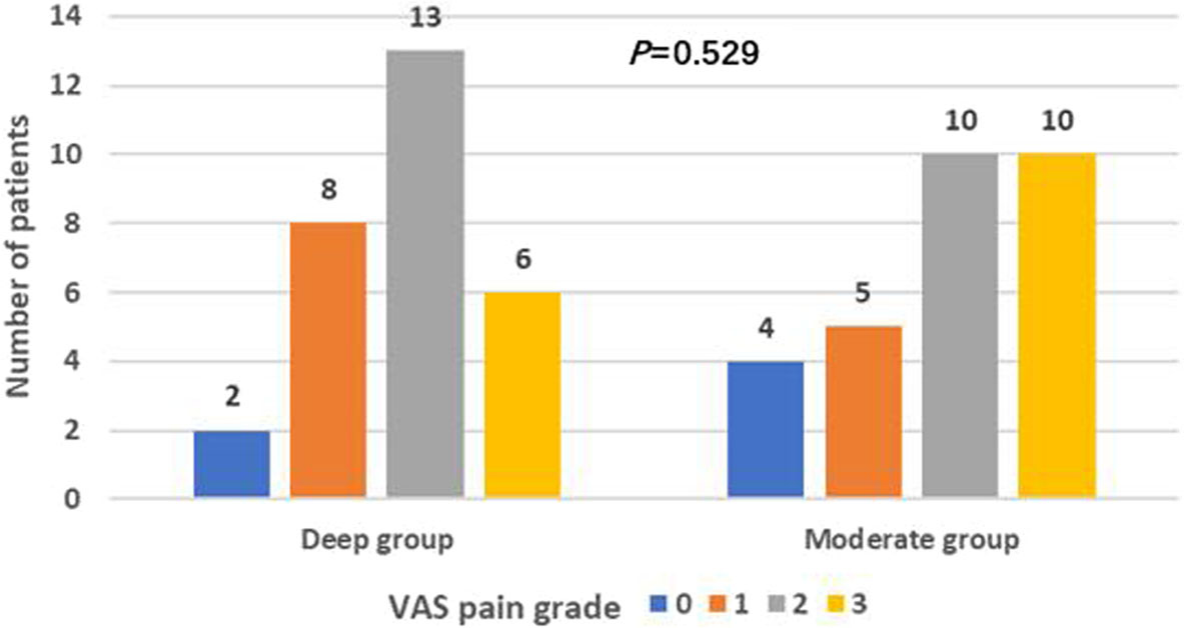
VAS pain grade. Note: Wilcoxon rank sum test, Z = − 0.63, *P* = 0.529

**Fig. 6:**
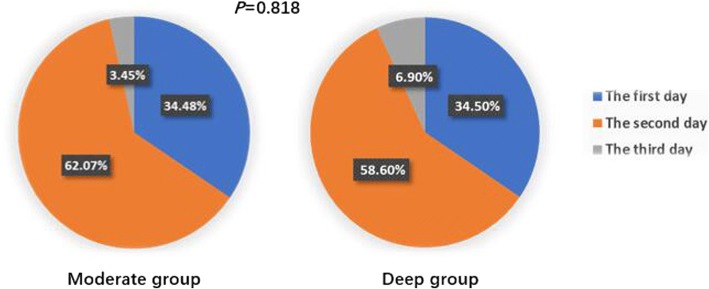
The time it took for patients to begin walking after surgery. Note: Student’s t-test, *P* = 0.818

The VAS pain grade in two groups were less than or equal to 3 (*P* = 0.529; Fig. 5). All patients began to get out of bed and walk within three days (*P* = 0.818; Fig. 6). The patients in both groups were hospitalized for an average of 5 days (*P* = 0.455; Fig. 4h), and no pulmonary complications occurred evaluated by doctor-in-charge during postoperative period.

## Discussion

This randomized controlled trial suggests that compared to moderate NMB, deep NMB can reduce the need for additional muscle relaxant and improve surgical conditions during thoracoscopic lobectomy. Deep NMB can provide these benefits while prolonging recovery time.

Under moderate NMB, up to 48% of patients in our study exhibited body movement, coughing, and spontaneous breathing during surgery. The main reason is that the surgery requires manipulation of the pulmonary arteriovenous vasculature and trachea as well as dissection of lymph nodes [21]. In addition, the surgical field in thoracoscopic lobectomy lies adjacent to the phrenic nerve, so inadequate muscle relaxation can allow diaphragm movement, which can cause coughing or spontaneous breathing, ultimately resulting in bleeding and other adverse consequences. Our results suggest that deep NMB can eliminate reactions caused by surgical stimulation and traction. These findings are consistent with previous work showing that deep NMB (PTC ≤ 1) can inhibit the diaphragm response to tracheal carina stimulation, significantly reducing the incidence of coughing and other adverse events [22, 23].

Since anesthesia depth can influence risk of body movement during surgery, we aimed to ensure anesthesia at a Narcotrend depth of 30 ± 5. This corresponds to anesthesia depth between E0 and E1, which lies within the ideal depth range for general surgery [24]. We chose to standardize anesthesia using the Narcotrend system because it is less sensitive than the BIS system to electromyographic activity and therefore more stable and reliable [25].

Our results suggest that deep NMB may be particularly useful for preventing contraction of muscles that normally recover quickly from relaxant therapy. During induction, 10.34% of patients exhibited coughing and diaphragm movement when the double lumen tube was inserted at TOF = 0. This indicates that adductor monitoring does not reflect the relaxation state of all muscles, and that the speed and degree of relaxation differ for different muscles. For example, the diaphragm relaxes more slowly and recovers more rapidly than the thumb adductor muscle [26]: when the adductor muscle was 90% inhibited in that study, the diaphragm was only 53–56% inhibited, and only 50% of the adductor muscle had recovered by the time the diaphragm showed complete recovery. This means that during intubation, the diaphragm may not be completely blocked and coughing may occur; during surgery, the preferential recovery of the diaphragm means that cough and other reactions are possible.

One concern with maintaining deep NMB during surgery is that it can prolong recovery time and increase the incidence of residual NMB. In our study, the deep NMB group was slower than the moderate NMB group by only 10 min in the time from drug withdrawal until recovery of TOF = 25%. This suggests that approximately 10 min are needed to achieve TOF 1–2 from PTC 1–5, consistent with previous work [27]. The two NMB groups in our study showed similar recovery index (time to recover TOF 25–75%), indicating that deep NMB did not affect patient recovery. This is consistent with the pharmacokinetic characteristics of cisatracurium [28], which shows no accumulation effect because its metabolite, N-methyltetrahydrocaproline, does not have muscle relaxant activities. As a result, time to recover from cisatracurium is dose-independent and predictable for patients of different ages. We found good correlation between PTC recovery and the first response to TOF stimulation (T1). This implies that neuromuscular recovery can be predicted and used to guide the rational use of muscle relaxant antagonists [29].

In both groups, patients showed normal pH, pCO_2_, and pO_2_ after extubation for 10 min; no serious respiratory acidosis (pCO_2_ > 55 mmHg) or hypoxemia (pO_2_ < 80 mmHg) occurred. All patients were able to get out of bed and walk within three days after surgery, the patients in both groups were hospitalized for an average of 5 days, and no pulmonary complications occurred. These results suggest that deep NMB does not substantially affect the prognosis of patients undergoing thoracoscopic lobectomy.

We found that in the deep NMB group, the average time for PTC to return to 1 after induction with 0.2 mg/kg cisatracurium was 43.33 ± 5.53 min, and the average time from pump stoppage (when PTC = 1–5) to recovery of TOF = 25% was 35.86 ± 6.50 min. These results indicate that an induction dosage of 0.2 mg/kg cisatracurium is reliably sufficient for 32 min (mean - 2SD) in ASA1–2 patients with BMI 18–25 kg/m^2^.

While we used cisatracurium in our study, because compared to other non-depolarizing muscle relaxants, cisatracurium released less histamine, its impact on the cardiovascular system is small and suitable for elderly patients. Rocuronium is also widely used and it behaves substantially differently. One study [30] reported the longest maintenance time to be 66 min and the shortest to be 25 min after induction with 0.6 mg/kg rocuronium, while the longest maintenance time was 44 min and shortest was 14 min after induction with other doses. That study attributed these different times to genetic and non-genetic factors such as age, sex, liver and kidney function. The disadvantages of rocuronium are compensated by Sugammadex [31], which can rapidly reverse moderate NMB at a dose of 2 mg/kg and deep NMB at a dose of 4 mg/kg [32]. Sugammadex can be suitable for many patients, even those with hepatic dysfunction, myasthenia gravis or morbid obesity [33].

Our study is one of the few to report continuous NMB monitoring. We ensured that the relative position of the palm and finger would not change when the patient was turned over (The patient had a lateral position with an outspread arm). As a result, we were able to ensure continuity in the calibration and comparability between patients. This may provide a superior approach to that of a previous study in which continuous pumping of muscle relaxants during thoracolaparoscopic esophagectomy was able to provide relatively stable surgical conditions but did not allow continuous monitoring of NMB [34].

Despite its strengths, our study has limitations, which should be taken into account when interpreting our results. We used the 4 point scale to evaluate surgical conditions, while this scale was adapted from a 5 point scale from Martini et al. and it seemingly had only been used in one study before and had not been validated, much more studies are needed to explore the validated scale for thoracic surgery. In addition, we used relatively wide range of 1 ≤ PTC ≤ 5 as the definition of deep NMB. Further study should compare surgical conditions under the two depths of 1 ≤ PTC ≤ 2 and 3 ≤ PTC ≤ 5, which can minimize the dosage range of muscle relaxants to meet surgical requirements. Since our study involved a small sample from a single center, our findings should be verified and extended in larger studies, preferably from multiple centers.

## Conclusions

Our study shows that deep NMB provides better surgical conditions for thoracoscopic lobectomy. It can reduce the need for additional muscle relaxant by surgeon and improve surgical conditions during thoracoscopic lobectomy. Deep NMB provide these benefits while prolonging recovery time.

## Abbreviations

NMB: Neuromuscular blockade
PTC: Post-tetanic count
TOF: Train of four
